# Elevation Pattern in Growth Coherency on the Southeastern Tibetan Plateau

**DOI:** 10.1371/journal.pone.0163201

**Published:** 2016-09-29

**Authors:** Lixin Lyu, Xu Deng, Qi-Bin Zhang

**Affiliations:** 1 State Key Laboratory of Vegetation and Environmental Change, Institute of Botany, Chinese Academy of Sciences, Beijing, 100093, China; 2 Graduate School of the Chinese Academy of Sciences, Beijing, 100093, China; Universite du Quebec a Chicoutimi, CANADA

## Abstract

It is generally expected that inter-annual changes in radial growth among trees would be similar to the increase in altitude due to the limitation of increasingly harsher climatic factors. Here, we examine whether this pattern exists in alpine forests on the southeastern Tibetan Plateau. Increment cores were collected from mature trees at the lower, middle and upper limits of balfour spruce (*Picea likiangensis* var. *balfouriana* (Rehd. et Wils.) Hillier ex Slsvin) forests at the Buze and Yela Mountains in Basu County, Changdu Prefecture of Tibet, China. The treeline elevations are 4320 m and 4510 m a.s.l. for Buze and Yela, respectively. Tree-ring widths were measured, crossdated, and detrended to obtain a sequence of ring-width indices for each individual sample. Annual growth rate, climate sensitivity, growth-climate relationships, and growth synchrony among trees were calculated and compared across altitudes. In Buze Mountain, the annual growth rate of trees has no significant difference across altitudes. The mean sensitivity of trees is lower at the treelines than at lower elevations. Tree growth has stronger correlation with winter temperature at upper elevations than at lower elevations, has significant correlation with moisture, not temperature, in the growing season, and the growth response to moisture is lower at the treeline than at lower elevations. The correlation among individual tree-ring sequences is lower at the treeline than at sites at lower elevation. In Yela Mountain, the characterisitics of annual growth rate, mean sensitivity, tree growth-climate relationships, and inter-serial correlation are similar to those in Buze, but their differences along altitudinal gradients are less significant as those in Buze. Our data do not support the general expectation of growth convergence among individuals with increasing altitude. We conclude that individual heterogeneity and microhabitat diversity are important features for treeline trees that may dampen the growth synchrony in trees. The results obtained in this study expand our knowledge about the pattern of forest growth along altitudinal gradients in high-elevation regions and demonstrate the importance of checking the growth of tree individuals before analyzing the average signal.

## Introduction

Patterns of tree growth along altitudinal gradients are fundamental for understanding the ecology of tree species and predicting the responses of forest ecosystem to climate change [1, 2]. Most studies investigated the altitudinal pattern in tree growth on population level by averaging the growth characteristics among trees [3–5]. However, variation in the growth of trees is ubiquitous in natural forests [6]. Understanding of such variation is essential to answering many important ecological questions, such as how synchronous trees will respond to a certain climatic factor with the increase in altitude [7, 8]. It is generally expected that inter-annual changes in radial growth among trees would be more similar to the increase in altitude due to the limitation of increasingly harsher climatic factors [9]. However, previous studies showed large variation in radial growth among trees at treelines on the Tibetan Plateau [10, 11].

Having an average elevation above 4000 m and an area greater than 2.5 million km^2^, the Tibetan Plateau is unique in its spatial characteristics of climate and forest. The Tibetan Plateau has typical mountain climatology and is the location of the highest treelines in the northern hemisphere [12]. Some studies showed that tree growth responds to moisture at lower elevations but responds to temperature at the treelines [13–15]. Other studies reported consistent growth responses to moisture along altitudinal gradients [16–19]. These conflicting results suggest that the pattern of tree growth along altitudinal gradients is complex, depending on regional climate regimes and/or forest types. The previous studies overwhelmingly treat forest stands as a uniform group of trees and mainly analyze the growth averages of different trees. Fewer studies explore growth variations among individuals across an altitudinal gradient. What remains unknown is whether trees would grow more synchronously with the increase in altitude regardless of the influence of temperature or moisture at the treelines.

Comparison of radial growth among trees along an altitudinal gradient is of importance for understanding the complexity of factors influencing treeline tree growth. If the growth characteristics among trees converge with the increase in altitude, then the treeline tree growth should be influenced by a dominant factor associated with altitudinal gradient. Otherwise, if such a convergence does not exist, the growth of treeline trees may be influenced by additional factors that may not be directly related to altitudinal changes. Such information about the variability of treeline tree growth at a fine scale (*i*.*e*., tree individuals) will provide important insights into treeline formation in general because treelines are not a straight line in essence but an ecotone consisting of trees.

The objective of this study is to test the general expectation of the growth convergence among trees at increasing altitude. We collected tree-ring samples at the lower, middle, and upper limits of spruce (*Picea likiangensis* var. *balfouriana* (Rehd. et Wils.) Hillier ex Slsvin) forests in two mountains of the southeastern Tibetan Plateau, and we examined the altitudinal patterns in growth rate, climate sensitivity, growth-climate relationships, and growth synchrony among trees. The methods used in this study are applicable to other alpine forests to test factors influencing tree growth along altitudinal gradients.

## Materials and Methods

### Study area and climate data

Spruce forests along altitudinal gradients in Buze Mountain (BZM, 30° 3.670′ N, 97° 15.31′ E) and Yela Mountain (YLM, 30°9.69′ N, 97°20.150′ E) were chosen for this study. These two mountains are located in Basu County of the southeastern Tibetan Plateau. The altitudinal belt of these forests ranges from 3750 to 4320 m a.s.l. in BZM and 4190 to 4510 m a.s.l. in YLM. The forest density generally decreases from the lower elevations to the treelines. The upper limit of the forests reached the alpine treeline (as judged by the treeline elevations in other surrounding summits), whereas the lower limit of the forests could not be the climatic lower limit, but determined by other factors such as landforms.

**Fig 1:**
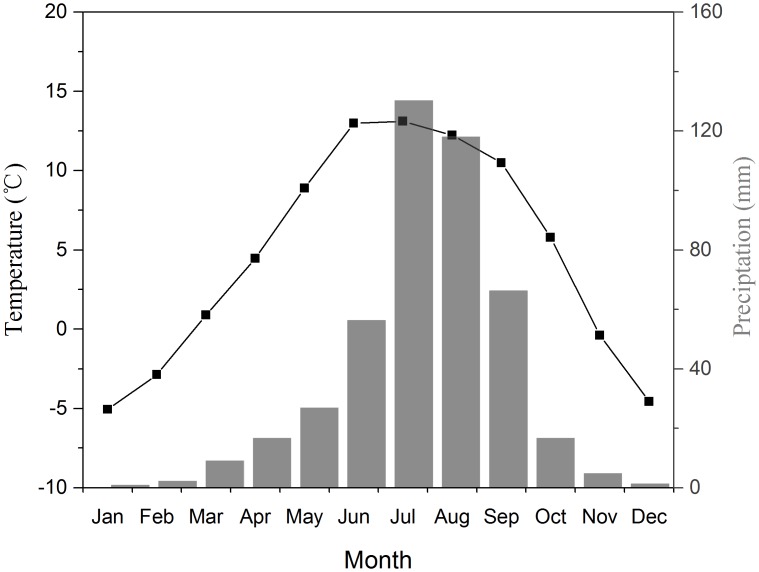
Mean monthly air temperature and monthly total precipitation for the period 1978–2010 from the Zuogong meteorological station.

The study area is characterized by the temperate continental climate, with hot-humid summers and cold-dry winters. Meteorological stations nearest to the sampling sites include Basu station (30°3′ N, 96°55′ E, 3260 m a.s.l.) and Zuogong station (29°40′ N, 97°50′ E, 3780 m a.s.l.). Because the monthly temperature (*r* = 0.997, *p* < 0.001) and precipitation records (*r* = 0.86, *p* < 0.001) between the two stations are highly correlated during the common period from 1991 to 2014, we used the climate data from Zuogong station (3780 m in elevation) to represent the climate variations of the study area for a longer record period (1978–2014) (Fig 1). Although the absolute values of climate in the meteorological station are different from those of the sampling sites due to the difference in elevation, the inter-annual variations of monthly mean temperature and total precipitation in low-elevation meteorological stations are highly correlated with those at the upper timberline on the southeastern Tibetan Plateau [20]. Therefore, the climate records are suitable for analysis of tree growth-climate relationships in this study. In addition to meteorological records, we also investigated the association between tree-ring growth and the monthly Palmer Drought Severity Index (PDSI), a measure of soil moisture conditions taking account of the influence of both precipitation and temperature [21]. The PDSI data for the grid (2.5 × 2.5 degree in latitude and longitude) covering our sampling sites were extracted from the global dataset over the period 1948–2002. The center coordinate of the PDSI grid is 32° 30′N, 96° 15′ E in this study.

### Tree-ring data

Since no endangered or protected species were involved in the sampling course, and the sampled forests neither belong to any private people/company nor covered by any nature reserve, no specific permissions were required for the field investigations. Nevertheless, we consulted the Forestry Bureau of Basu County on the treeline formations and the road conditions. Increment cores (one core per tree) were collected from mature trees at breast height and in a direction parallel to the height contour of the mountain. Thirty-one, 33 and 33 cores were collected from the lower, middle, and upper limits of the forest in BZM, respectively. Likewise, thirty-eight, 31 and 26 cores were collected in YLM (Table 1). These cores were mounted on grooved wooden boards and polished to make the ring boundaries clearly visible. Ring widths were measured using a Lintab tree-ring measurement system to 0.001 mm precision. All samples were crossdated following dendrochronological techniques so that each ring was assigned the calendar year of its formation [22]. The quality of crossdating was checked using the program COFECHA [23].

**Table 1:** Information of the study sites and ring-width series of *Picea likiangensis var*. *balfouriana* (Rehd. et Wils.) Hillier ex Slsvin.

Sites	Altitude (m a.s.l.)	Slope aspect	Canopy coverage (%)	Number of cores	Chronology time span	Mean inter-series correlation	SD	MS
BZM: Upper	4320	NE	40	33	1750–2010	0.79	0.23	0.18
BZM: Middle	4110	NE	50	33	1565–2010	0.77	0.4	0.42
BZM: Lower	3750	NE	55	31	1795–2010	0.8	0.48	0.46
YLM: Upper	4510	E	35	26	1865–2010	0.54	0.23	0.16
YLM: Middle	4370	E	50	31	1880–2010	0.6	0.27	0.18
YLM: Lower	4190	E	60	38	1960–2010	0.53	0.33	0.21

Note: SD: standard deviation in tree rings; MS: mean sensitivity in tree rings.

The measured ring-width sequence of each individual tree was standardized using the program ARSTAN [24], in which a 128-year spline with a frequency cutoff at 50% of the sequence length was applied to remove the growth trend related to tree age and stand dynamics [25]. The 128-year spine is the result of the tradeoff between the stiffness (longer time window; e.g., 200-year spline; some of the age effects would remain) and signal loss due to overfitting (shorter time window; e.g., 50-year spline; part of the low frequency climate signals would be lost). The resultant sequence of ring-width indices for each individual tree reflects the growth variation in response to changes in fine-scale habitat conditions. These data were used for comparison of growth patterns along an altitudinal gradient. Site tree-ring chronologies were also developed by averaging the indices of individual ring-width sequences for each elevation in BZM and YLM. These chronologies were used to identify the stand level growth-climate relationships at different elevations.

### Data analysis

To compare patterns of tree-ring growth along the altitudinal gradient, we conducted the following four types of calculations.

#### Mean annual growth rate

Tree-ring width sequences usually exhibit a trend that is non-climatic in origin but related to changes in size with tree aging. To remove such age effects, we calculated the basal area increment (BAI) of each ring for cores that passed through or close to pith and averaged these BAI values to obtain the mean annual growth rate for each sample [4, 5, 26]. These mean annual growth rates were then compared among trees of similar age at different elevations.

#### Mean tree-ring sensitivity

The degree of year-to-year change in tree-ring widths was evaluated by calculating the mean sensitivity of tree rings for each sample following the formula below [22].

$$\text{Mean}\;\text{sensitivity}=\frac{1}{n-1}\sum_{i=2}^{n}\frac{2|r_{i}-r_{i-1}|}{r_{i}+r_{i-1}}$$ (1)

where *i* represents the *i*th ring, and n is the total number of rings. The value of mean sensitivity ranges from zero to two. The mean sensitivity is zero if there is no difference among the tree rings, and the mean sensitivity is two if the tree grows in one year and stops growth in the next.

#### Growth-climate relationships

Pearson correlation coefficients between site tree-ring chronologies and monthly mean temperature, total monthly precipitation and PDSI were calculated for stands of each elevation. Seasonal climatic factors that likely had an influence on tree growth were selected for further correlations between the seasonal climate and growth of trees at each elevation.

#### Tree-ring synchrony

Inter-series correlation coefficients were calculated among individual tree-ring indices to indicate the degree to which trees respond to environmental change synchronously.

In contrast with traditional dendroecological methods that emphasize analysis of site chronologies, we address the abovementioned four parameters based on tree individuals to obtain fine-scale information. Comparisons of these parameters among sites of different elevations allow examining the general expectation that growth converges as altitude increase in the area under study.

## Results

### Tree-ring width chronologies along the altitudinal gradients

**Fig 2:**
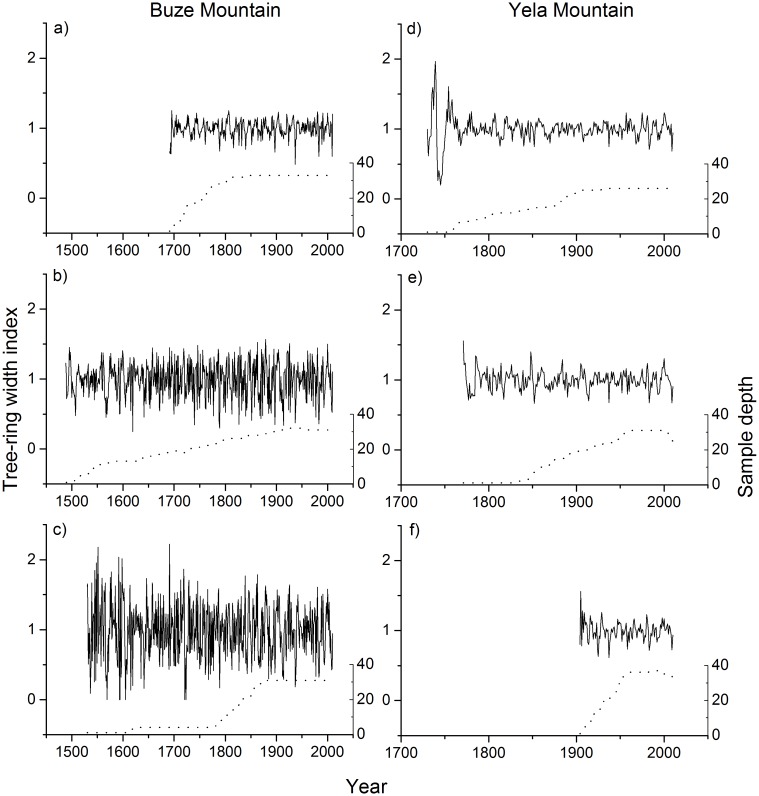
Tree-ring chronologies (solid line) for Buze Mountain (left panel) and Yela Mountain (right panel). a/d, b/e and c/f represent the upper, middle and lower elevation sites respectively. The dashed lines represent the numbers of tree-ring samples.

A total number of 192 samples were successfully crossdated (Table 1). The age ranges of the samples at the upper, middle and lower elevation sites are 170–320, 85–523 and 130–481 years in BZM, and 82–281, 52–240 and 28–107 years in YLM. Site chronologies were developed for different elevations in BZM and YLM (Fig 2, Table 2). The chronology intervals with an expressed population signal (a measure of the amount of common signals in tree rings) greater than 0.85 were 1575–2010, 1565–2010 and 1795–2010 for the upper, middle and lower elevation sites in BZM, and were 1865–2010, 1880–2010 and 1960–2010 in YLM. Moreover, the large percentage of variance explained by the first eigenvectors over the common period indicates that common signals are strong among trees in each forest stand. Additionally, autocorrelations at treelines were higher than at lower elevations in both transects.

**Table 2:** Statistical characteristics of the standard tree-ring width chronology for each study site.

Site chronology	MSL	EPS^#^	SNR^#^	VFE^#^ (%)	AC_1_
BZM: Upper	260	0.96	27.02	51.2	0.53
BZM: Middle	338	0.97	30.90	54.6	0.25
BZM: Lower	189	0.99	70.65	72.0	0.32
YLM: Upper	176	0.93	12.36	38.9	0.74
YLM: Middle	122	0.92	10.95	38.4	0.69
YLM: Lower	76	0.89	7.67	26.4	0.34

^#^ Stats calculated over the common period 1961–2010.

Note: MSL means mean segment length, EPS means expressed population signal, SNR means signal-to-noise ratio, VFE means the percentage of variance explained by the first eigenvector.

### Mean annual growth rate along altitudinal gradients

**Fig 3:**
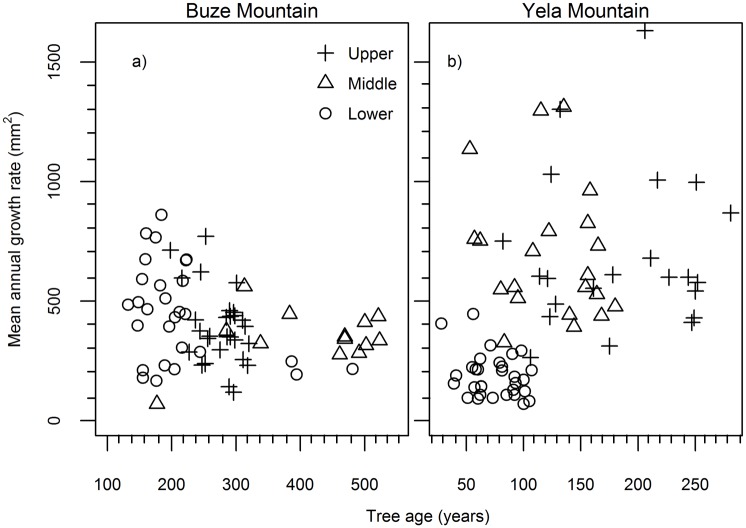
Mean annual growth rate (represented by basal area increment) of trees at three altitudes in Buze and Yela Mountains.

**Fig 4:**
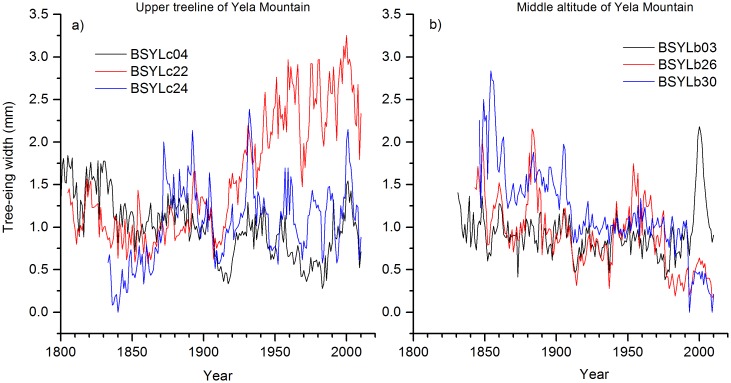
Fast radial growth in tree number 22 at the treeline (a), and examples of different growth patterns among trees at the treeline and mid-elevation sites (b) in Yela Mountain.

There is no discernable altitudinal difference in the mean annual growth rate in trees of similar age in BZM and YLM (Fig 3). The greatest mean annual growth rate occurs in a 206-years-old tree located at the upper elevation site in YLM. This tree showed an increased radial growth since the 1910s and remained at a high level of annual growth rate since the 1950s. This occurred despite a brief growth reduction around 1970 (Fig 4a). This increased growth is, however, not a general pattern in other trees. For example, such a growth trend did not exist in the trees of closest age (two trees) at the same elevation (Fig 4a) or in the trees of nearest age (three trees) at the middle elevation in YLM (Fig 4b). Note that the tree ages of the lower altitude of YLM were clearly lower than those of the upper two altitudes. The tree growth data (BAI) of the lower elevation in YLM was thus not used to infer tree growth pattern along the altitudinal gradient due to potential age effects on mean growth rates.

### Growth coherency along the altitudinal gradients

**Fig 5:**
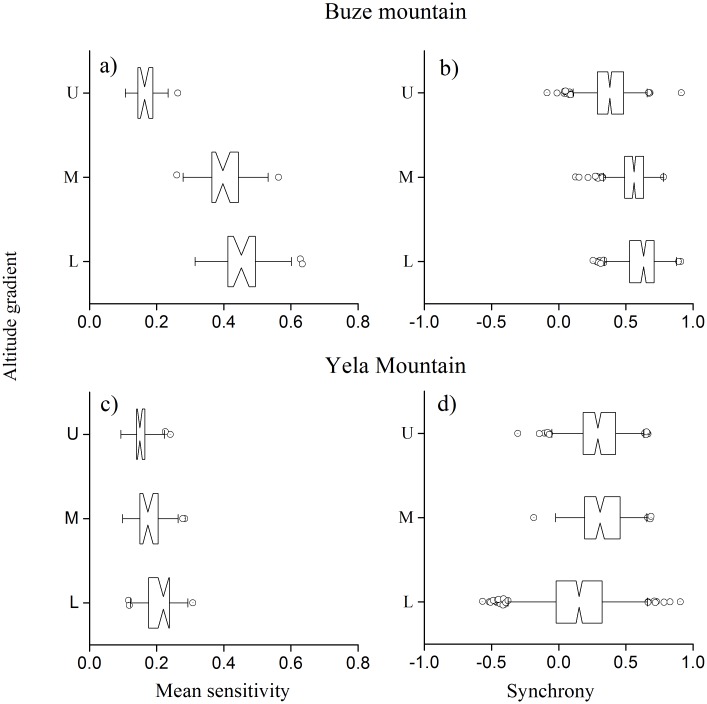
Mean sensitivity and growth synchrony among individual tree-ring sequences at three altitudes in Buze and Yela Mountains. L, M, and U represent lower, middle and upper elevation sites.

All samples are sensitive to inter-annual environmental variations (Fig 5a and 5b). In BZM, the treeline trees had significantly lower sensitivity than those at sites of lower elevation. In YLM, the tree-ring sensitivity is lower than that in BZM. Similar to BZM, treeline trees in YLM had lower sensitivity compared with those at sites of lower elevation.

Growth synchrony among trees at sites of different elevations in the two mountains showed a pattern similar to growth sensitivity (Fig 5c and 5d). Tree growth generally had higher synchrony in BZM than YLM. The synchrony in treeline trees is significantly lower (*p* < 0.001) than that of the lower elevations in BZM, whereas it had no significant difference along the altitudinal gradient in YLM.

### Tree growth—climate relationships at tree and population level

**Fig 6:**
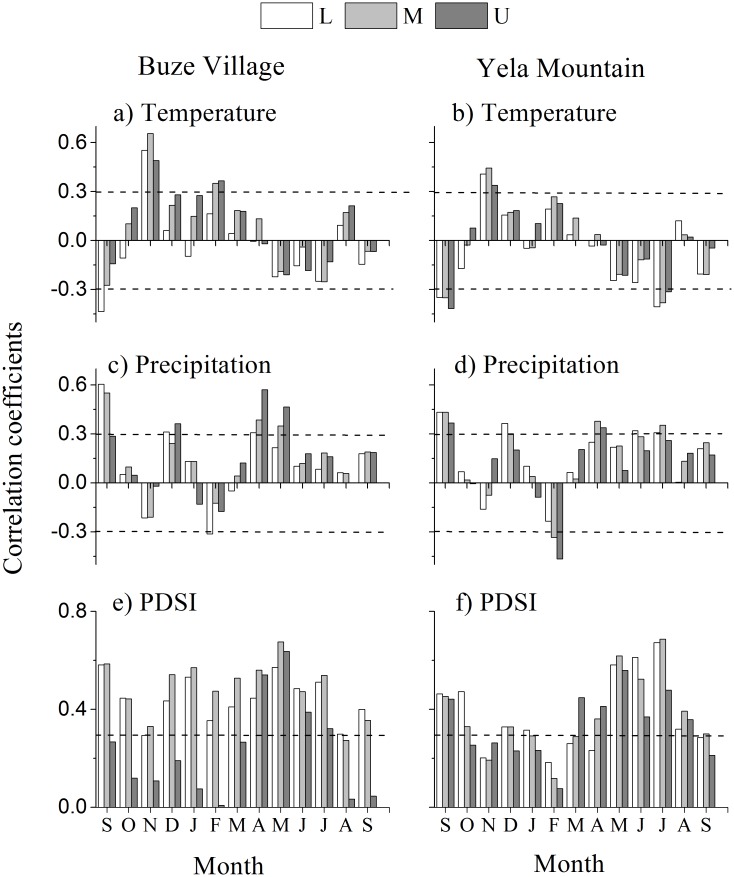
Correlation coefficients between the tree-ring chronologies of different elevation and monthly climatic factors in Buza and Yela Mountains. Monthly climatic factors are from September of the prior growth year to September of the current growth year. The dotted lines represent significance at the level of *p* < 0.05. L, M, and U represent the lower, middle and upper elevations.

**Fig 7:**
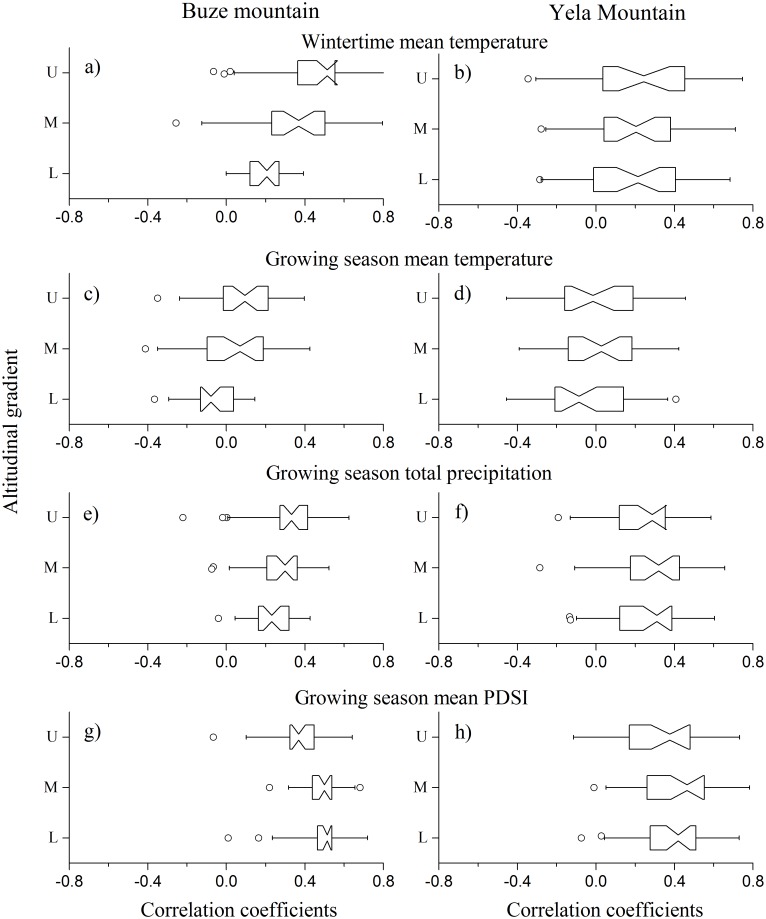
Box plot of the correlation coefficients between the climate data and growth indices of trees in Buze and Yela Mountains. The position of the notch represents the median value; the right and left edges of the box represent the 1st and 3rd quartiles; circles outside of the line represent outliers (beyond 1.5 interquartile range of the box). If two notch boxes do not overlap, this indicates a statistically significant difference between the two medians. L, M and U represent the lower, middle and upper elevations.

Analysis of the relationships between site tree-ring chronologies and climate showed that the chronologies were positively correlated with two seasonal factors: winter (previous November to current February) temperature and growing season (April to September) moisture (Fig 6). To examine the fine-scale growth-climate response, we calculated the correlation coefficients between these seasonal climate factors and the ring-width indices of trees for each site. The results showed that correlation coefficients between tree growth and climate had greater variability among trees in YLM than in BZM and, in both mountains, tree rings had greater correlation coefficients with the growing season moisture than winter temperature with only one exception in the treeline at BZM (Fig 7). In addition, the growth-moisture relationships had no significant difference at different elevations for the two mountains (Fig 7), yet growth-temperature relationships strengthen with the increase in elevation in BZM and had no difference in YLM.

## Discussion

### Do treeline trees respond to climate differently than trees at lower elevation?

It is widely reported that trees at lower elevation respond to moisture, whereas trees at treelines respond to temperature [2, 15, 27]. Our study shows that there is no appreciable change in climate-growth relationship along the altitudinal gradients. Tree growth is positively correlated with growing-season moisture and winter temperature across the altitudinal gradient, although there is a slight difference in the strength of the response. We believe that this phenomenon is a typical characteristic of the alpine forest ecology in this region [18].

The Tibetan plateau has a strong radiation load in the growing season due to the mass elevation effect [28, 29]. Based on the temperature lapse rate on the southeastern Tibetan Plateau [30], the growing season mean temperatures are about 7.8°C at the treeline in YLM and 9°C at the treeline in BZM. These temperatures are above the global treeline temperature threshold of 6.7±0.8°C [31], which may be a reason why the growing season temperature did not limit treeline tree growth in our two study sites. The follow-up question is why trees stop moving upslope to a higher position. Here, we suspect that moisture deficiency may be a factor limiting seed germination and/or the survival of seedlings at higher elevations [32, 33]. Although precipitation mostly falls during the growing season, the seasonal evapotranspiration is also the highest, causing the growth of trees to suffer from the shortage of moisture [34]. In addition to high radiation load, strengthened wind speed and reduced vegetation cover at open treeline ecotones could also increase surface evapotranspiration rates [35–37]. The response of treeline trees to moisture deficiency was also reported in many study sites on the Tibetan Plateau [17–19, 32, 38–41].

### Do trees grow slower, more sensitively and synchronously among individuals with the increase in altitude?

According to reports in the literature, the answers to the above questions should be yes they do, because the growth of trees will be limited by increasingly harsher environmental factors with the increase in altitude [36, 42, 43]. However, our results showed unexpected patterns of radial growth among trees along the altitudinal gradient. This result suggests that individual differences and microhabitat diversity are underestimated in terms of their role in regulating tree growth at high elevations.

The pattern of mean annual growth rate in trees and the sensitivity of tree rings along altitudinal gradients suggest that treeline trees may have developed adaptive functional traits in their growth history to cope with climatic stresses. Studies of *Picea likiangensis* (Franchet) E. Pritzel and *P*. *asperata* Masters in the eastern Himalaya showed that these trees had a lower ratio of foliage area/stem cross-sectional area, a lower ratio of root mass/foliage area, a smaller specific leaf area, and less negative δ^13^C at sites at upper elevations than at lower elevations, indicating apparent acclimation to drought at higher elevations [44]. On the northeastern Tibetan Plateau, the stomatal density and length and dry weight of needles in *Picea crassifolia* decreased with increasing altitude above 3000 m to adapt to drought conditions [45]. In addition, the reduced population density at treelines is also favorable for tree growth by decreasing competition among trees [46].

In addition to the acclimation of trees, an alternative explanation is that the limiting factor for tree growth has less year-to-year variability at treelines than at lower elevation sites. The elevation-dependent distribution of clouds and fog in the mountainous forests could be a contributing factor. Previous studies showed that solar radiation in summer is either no different or lower at treelines compared with lower elevations due to the effect of clouds and fog [2, 36]. On the Tibetan Plateau, clouds and fog aloft treelines are more frequent and stable than at lower elevations [47]. Thus, annual change in climate conditions may be dampened at the treelines.

Our study shows decreased synchrony in treeline tree growth at BZM and little difference in growth synchrony among sites of different elevation at YLM. Between-site differences in growth synchrony could be attributable to site differences in altitudinal range, slope aspect and tree ages in this study. These observations suggest that treeline tree growth either has large differences among individuals or is more influenced by microhabitat conditions than sites of lower elevation. Genetic differences were considered as a possible factor causing divergent growth-climate response among trees at *Picea crassifolia* treelines in the northeastern Tibetan Plateau [10]. There are many reports about the importance of treeline microhabitat in buffering regional climatic stress, thus influencing tree mortality and growth [35, 37, 48, 49]. In addition to climate, other factors such as disturbances [50–52], soil nutrition availability [53] and biotic interactions [27, 54, 55] could create a multitude of microhabitats, altering the growth response at treelines and leading to more idiosyncratic tree rings than those at low elevation. To identify which explanation is valid for the altitudinal pattern of tree growth synchrony, *in situ* observation of both climate conditions and eco-physiological features is required. Here, we did not explicitly investigate which factor was more responsible for growth changes. Rather, we demonstrate that there is a possibility that the factors limiting tree growth from low to high elevation may not converge to a dominating single factor.

## Conclusions

Our study of *P*. *likiangensis* forests along the altitudinal gradients at two mountains of the southeastern Tibetan Plateau demonstrates that the growth of these alpine forests are mainly under moisture control, and treeline trees may not grow more slowly, sensitively and synchronously among individuals compared with those at sites of lower elevation. These observations suggest that growth-limiting factors may not necessarily converge with the increase in altitude. Treeline tree growth is more likely related to heterogeneity (e.g., functional traits, health status, and genetics) and could be influenced by a multitude of microhabitat stresses and disturbances. Our results imply that, in dendrochronological reconstruction of past climate, ring widths of treeline trees may not always contain stronger regional climate signals, and a large number of samples may be needed to reflect the true common signals. In terms of future growth prediction, if growing season droughts occur due to temperature increase [56], trees at lower elevation will possibly reduce growth accordingly, whereas treeline trees will respond in a more complicated way depending upon an interaction of temperature, moisture, individual sensitivity and heterogeneity of microhabitat conditions. The results obtained in this study expand our knowledge about the pattern of forest growth along altitudinal gradients in alpine environments and address the importance of examining the growth of trees when averaging them for common signal analyses.
